# Chronic Eosinophilic Pneumonia: A Pneumopathy With Many Faces

**DOI:** 10.7759/cureus.111192

**Published:** 2026-06-20

**Authors:** Chaynez Rachid, Lina Romane, Mohamed Ijim, Oussama Fikri, Lamyae Amro

**Affiliations:** 1 Pulmonology Department, Arrazi Hospital, Mohammed VI University Hospital Centre (CHU Mohammed VI), Marrakech, MAR; 2 Pulmonology Department, Morpho-Sciences Research Laboratory (LRMS), Faculty of Medicine and Pharmacy of Marrakech (FMPM), Cadi Ayyad University (UCA), Marrakech, MAR

**Keywords:** alveolar opacities, carrington's disease, chronic eosinophilic pneumonia, corticosteroids, pulmonary eosinophilia

## Abstract

Chronic eosinophilic pneumonia (CEP), also known as Carrington's disease, is a rare idiopathic eosinophilic lung disorder characterized by eosinophilic infiltration of the pulmonary parenchyma and alveolar spaces. It typically presents with subacute respiratory and constitutional symptoms, including cough, dyspnea, fever, and weight loss, and can mimic infectious pneumonia or pulmonary tuberculosis, making diagnosis challenging.

We report the case of a 40-year-old woman who presented with a 40-day history of progressively worsening dyspnea, persistent cough, subjective fever, and deterioration of her general condition. Chest imaging revealed bilateral alveolar-interstitial infiltrates, while investigations for tuberculosis were negative. Further diagnostic evaluation established the diagnosis of CEP. The patient was treated with systemic corticosteroids, resulting in rapid clinical and radiological improvement and a favorable outcome on follow-up. This case highlights the importance of considering CEP in the differential diagnosis of diffuse pulmonary infiltrates and emphasizes the remarkable responsiveness of the disease to corticosteroid therapy.

## Introduction

Chronic eosinophilic pneumonia (CEP), first characterized by Carrington et al. in 1969, is a rare idiopathic disorder characterized by the massive accumulation of eosinophils within the pulmonary parenchyma and alveolar spaces. While the exact pathogenesis remains elusive, it is frequently associated with an underlying type 2 (Th2) immune response, often manifesting in patients with a pre-existing history of adult-onset asthma or atopic rhinitis [1]. Clinically, CEP presents with a subacute progression of constitutional symptoms, including cough, dyspnea, night sweats, and weight loss. Radiologically, the condition is famously associated with "peripheral opacities," often described as the "photographic negative of pulmonary edema," a hallmark that, while classic, is not present in all cases [2]. The diagnosis relies on the triad of clinical symptoms, peripheral blood eosinophilia, and, crucially, high eosinophil counts in bronchoalveolar lavage (BAL) fluid, typically exceeding 25%. While the response to systemic corticosteroids is characteristically rapid and dramatic, the clinical course is often complicated by a high rate of relapse upon steroid tapering, necessitating long-term management and, in some instances, the consideration of steroid-sparing biologic agents [3]. This case report describes a 40-year-old woman presenting with Modified Medical Research Council (mMRC) grade 4 dyspnea, highlighting the diagnostic difficulty, and discusses the current therapeutic challenges associated with long-term eosinophilic control.

## Case presentation

We present the case of a 40-year-old woman who had been followed for neurosis for one month. She had never received treatment for tuberculosis and reported no recent contact with individuals with tuberculosis. She had no history of chronic dyspnea or chronic bronchitis, was not exposed to any specific environmental risk factors, and had no personal or family history of atopy.

The patient was admitted for etiological investigation of a diffuse interstitial lung disease. Her symptoms began 40 days before admission, following an episode of exposure to cold weather, with exertional dyspnea associated with a persistent dry cough. The clinical course was marked by progressive worsening of dyspnea, reaching Sadoul stage V, accompanied by a productive cough with whitish sputum, without chest pain or other thoracic or extrathoracic symptoms. These manifestations occurred in a context of subjective fever and deterioration of her general condition, including profound asthenia, anorexia, and unquantified weight loss.

The patient initially consulted a general practitioner, who requested a tuberculosis workup that proved negative and prescribed amoxicillin/clavulanic acid for one week. She also underwent a chest computed tomography (CT) scan one month later, which revealed bilateral basal infiltrates. Due to the persistence of the same symptoms, she was referred to the pulmonary emergency department, where she was hospitalized for further evaluation and management of CEP.

Clinical examination revealed a conscious patient with a Glasgow Coma Scale score of 15/15, a World Health Organization (WHO) performance status of 1, a respiratory rate of 20 breaths/min, an oxygen saturation of 96% on room air, and a heart rate of 84 beats/min. Pleuropulmonary examination revealed mild bibasal crackles. A posteroanterior chest radiograph demonstrated an alveolar-interstitial syndrome with bilateral infiltrative opacities (Figure 1).

**Figure 1:**
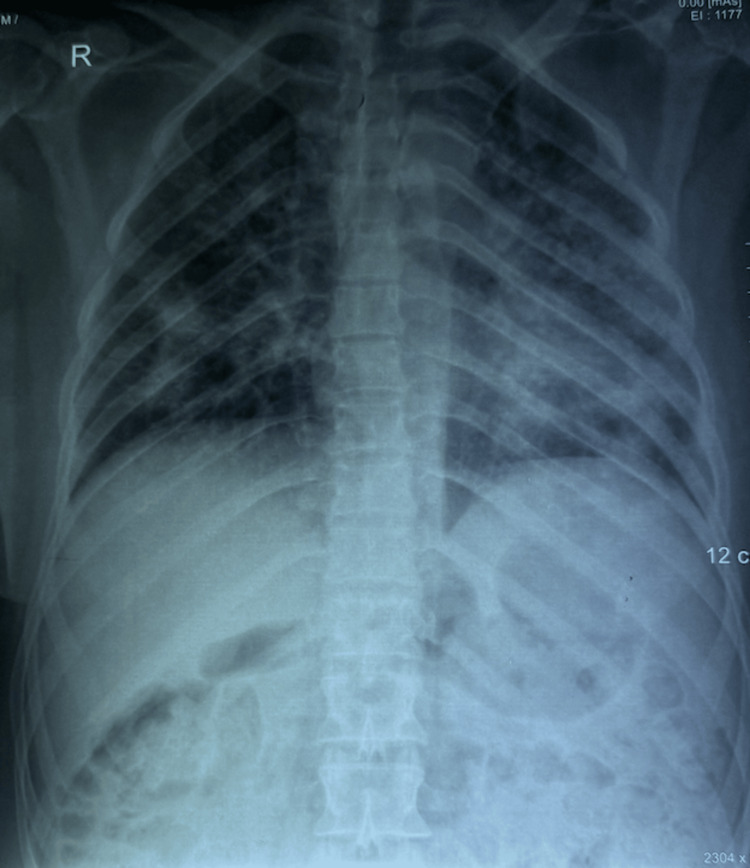
Posteroanterior chest radiograph demonstrating bilateral interstitial syndrome with foci condensation The image shows diffuse bilateral infiltrative opacities.

Laboratory investigations revealed leukocytosis of 14,290/mm³, marked peripheral blood eosinophilia of 7560/mm³, a C-reactive protein (CRP) level of 101 mg/L, and an elevated total immunoglobulin E (IgE) level of 419 IU/mL. Antineutrophil cytoplasmic antibodies (ANCA) were negative (Table 1). Sputum examination for mycobacteria was negative. 

**Table 1:** Eosinophilia workup: key laboratory findings ANCA: antineutrophil cytoplasmic antibody; IgE: immunoglobulin E

Test	Result	Reference range
Eosinophil count	7560/mm³	0-500/mm³
C-reactive protein	101 mg/L	<5 mg/L
Total IgE	419 IU/mL	0-150 IU/mL
ANCA (p-ANCA/c-ANCA)	Negative	<20

Chest CT demonstrated multiple areas of consolidation and scattered ground-glass opacities, predominantly peripheral in distribution, associated with septal reticulations, resulting in a crazy-paving pattern involving the left lower lobe (LLL) (Figure 2).

**Figure 2:**
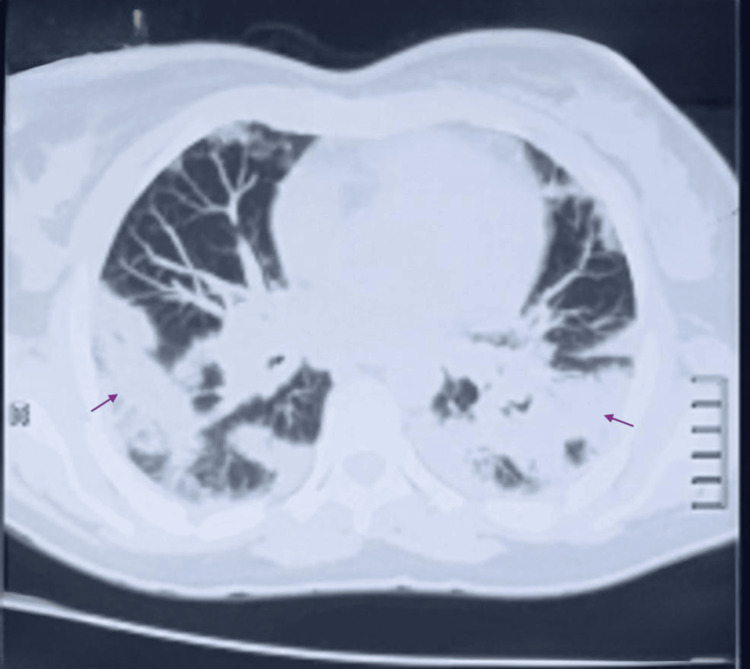
Representative axial chest computed tomography slices showing bilateral subpleural areas of consolidation The consolidation predominantly affects the lower lobes (arrows), a radiologic pattern suggestive of chronic eosinophilic pneumonia in the appropriate clinical context. Differential diagnoses were considered and excluded.

The diagnosis of CEP (Carrington's disease) was established based on the presence of compatible clinical and radiological features, marked peripheral blood eosinophilia, elevated serum IgE levels, exclusion of infectious causes, and characteristic radiological pattern of peripheral pulmonary infiltrates. 

Given the severity of dyspnea (mMRC grade 4/Sadoul stage V), extensive bilateral radiologic involvement, and marked eosinophilia, high-dose intravenous corticosteroid therapy with methylprednisolone (1,000 mg/day for three consecutive days) was initiated, followed by oral prednisone (1 mg/kg/day). Following this high-dose corticosteroid regimen, asthenia and cough resolved, and oxygen saturation improved. Once the definitive diagnosis of CEP was confirmed, oral corticosteroid therapy was continued with gradual tapering according to clinical and radiological response.

Follow-up chest CT demonstrated marked radiological improvement, with resolution of the previous abnormalities and persistence of a residual arc-shaped ground-glass opacity (Figure 3). The subsequent clinical course was favorable, with significant improvement in symptoms.

**Figure 3:**
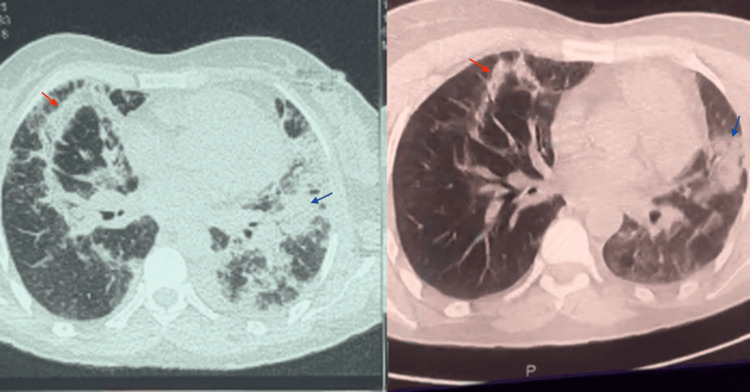
Representative axial chest computed tomography images obtained after two weeks of systemic corticosteroid therapy Compared with the initial examination, there is marked regression of the bilateral peripheral and subpleural consolidations previously observed. These findings demonstrate a near-complete radiological response to corticosteroid treatment, consistent with the characteristic rapid improvement seen in chronic eosinophilic pneumonia.

## Discussion

CEP, historically known as Carrington's disease, is a distinct clinicopathological entity characterized by eosinophilic infiltration of the pulmonary parenchyma in the absence of an identifiable cause. Although CEP has occasionally been considered a localized pulmonary variant of idiopathic hypereosinophilic syndrome (HES), it is distinguished by its exclusive involvement of the lungs [4].

CEP primarily affects middle-aged adults, with the highest incidence reported between 40 and 50 years of age. Pediatric cases are rare but have been described. The disease typically presents with a subacute or chronic clinical course marked by respiratory symptoms, including persistent cough and progressive dyspnea, which may be associated with wheezing and initially mistaken for asthma. Constitutional manifestations such as fatigue, malaise, and low-grade fever are also common. Owing to the nonspecific nature of these symptoms, diagnosis is frequently delayed, with an average interval of four to six months between symptom onset and diagnosis [5].

The absence of extrapulmonary involvement represents a key diagnostic criterion and helps distinguish CEP from other eosinophilic disorders, particularly eosinophilic granulomatosis with polyangiitis (EGPA) and systemic vasculitides. The differential diagnosis encompasses both primary and secondary causes of pulmonary eosinophilia. Primary disorders include acute eosinophilic pneumonia, CEP, EGPA, and chronic idiopathic HES. Secondary causes comprise parasitic infections such as ascariasis, tropical pulmonary eosinophilia, and strongyloidiasis; non-parasitic infections including coccidioidomycosis and tuberculosis; allergic bronchopulmonary mycoses and aspergillosis; as well as drug-, toxin-, and radiation-induced eosinophilic pneumonitis. Additional pulmonary conditions occasionally associated with eosinophilia include cryptogenic organizing pneumonia, interstitial pulmonary fibrosis, pulmonary Langerhans cell histiocytosis, sarcoidosis, granulomatosis with polyangiitis, malignancy-associated eosinophilia, and transplant rejection [6].

Thoracic imaging, particularly high-resolution computed tomography (HRCT), plays a central role in the diagnostic evaluation. Characteristic radiological findings include peripheral, often migratory, areas of ground-glass opacity and alveolar consolidation, predominantly involving the upper and middle lung fields. A notable feature of these lesions is their rapid regression following corticosteroid therapy.

Peripheral blood eosinophilia exceeding 500 cells/mm³ is highly suggestive of the diagnosis, although it may be absent in approximately 10% of patients. BAL is a cornerstone of the diagnostic workup and typically reveals eosinophilic alveolitis, with eosinophils accounting for approximately 25-40% of recovered cells.

Although pulmonary function tests are not required for diagnosis, they provide valuable information for baseline assessment and longitudinal follow-up, particularly in patients with concomitant asthma. Spirometry may demonstrate either obstructive or restrictive ventilatory defects. Early eosinophilic infiltration of the airways is frequently associated with bronchial obstruction. Arterial blood gas analysis generally reveals mild hypoxemia, contrasting with acute eosinophilic pneumonia, in which severe hypoxemia and respiratory failure are more common [7].

Histopathological confirmation is not routinely necessary. When lung biopsy is performed, the characteristic findings include eosinophilic and lymphocytic infiltration of the alveolar spaces and interstitium, without evidence of granulomatous inflammation or vasculitis.

The diagnosis of CEP is based on four major criteria: (1) subacute or chronic respiratory symptoms, often accompanied by constitutional manifestations; (2) peripheral blood and/or alveolar eosinophilia; (3) predominantly alveolar pulmonary infiltrates on imaging; and (4) exclusion of alternative causes of eosinophilic lung disease.

Systemic corticosteroids remain the first-line treatment, with recommended initial prednisone-equivalent doses ranging from 0.5 to 1 mg/kg/day. The therapeutic response is typically rapid and dramatic, often supporting the diagnosis retrospectively. Clinical improvement usually occurs within days, and complete radiological resolution may be observed within two weeks of treatment initiation [8].

Despite the excellent initial response to corticosteroid therapy, the long-term course is frequently marked by relapses, particularly during dose reduction or treatment withdrawal. Recurrence rates may reach 50%, and prolonged corticosteroid therapy extending beyond one year is required in more than two-thirds of patients. Although uncommon, progression to pulmonary fibrosis has been reported, underscoring the need for long-term follow-up. Spontaneous remission remains rare, occurring in approximately 10% of cases [9].

## Conclusions

CEP, or Carrington's disease, remains a rare interstitial lung pathology that is essential to identify early to avoid prolonged diagnostic wandering. Although its clinical and radiological presentation may initially mimic infectious pneumonia, the persistence of symptoms under antibiotic therapy and the presence of hypereosinophilia must imperatively guide the clinician toward this etiology. The short-term prognosis is excellent due to a spectacular sensitivity to corticosteroids, a true "ex juvantibus" diagnostic test. However, the major challenge lies in the long-term management of the high risk of relapse and steroid dependency, which significantly impact patients' quality of life. The close association between CEP and severe asthma highlights the importance of a comprehensive phenotypic approach to Th2 inflammation. In the future, the early integration of biologic therapies (anti-IL-5 or anti-IL-4/IL-13) could transform the management of CEP by offering an effective steroid-sparing alternative, thereby reducing iatrogenic comorbidities while maintaining durable remission.
